# Limits to molecular matched-pair analysis: the experimental uncertainty case

**DOI:** 10.1186/1758-2946-6-S1-O6

**Published:** 2014-03-11

**Authors:** Christian Kramer, Klaus Liedl

**Affiliations:** 1 Department of General, Inorganic and Theoretical Chemistry, University of Innsbruck, 6020 Innsbruck, Austria

## 

Matched-Molecular Pair (MMP) analysis has recently emerged as a data analysis technique in medicinal chemistry. It quickly gained scientific momentum because it tackles key questions in lead optimization. In contrast to classical global QSAR models that attempt to predict the absolute numbers of ADME (absorption, distribution, metabolism, excretion) and toxicological properties, MMP analyses predict the difference in (bio-) chemical properties that can be expected due to small chemical modifications to lead structures, with a much smaller and well-controlled error than global QSAR models.

The power of MMP analysis depends on the number of previously documented similar molecular transformations, whereas the definition of chemical similarity plays a key role: the more generous the definition of similarity of the anchoring region, the more examples are available. The more strict the definition of similarity, the lower the variability and thus the clearer the effect on ADME-Tox parameters, but also the less data pairs will be available [1].

The (bio-) chemical effect and the significance of the results depends on the experimental uncertainty (=noise) in the data. There is a clear mathematical association between the noise level and the minimum activity difference necessary for statistical significance. Here we demonstrate how the experimental uncertainty and variability[2,3] affect Matched Molecular Pair Analysis. It can be shown that for small sample sizes (Context-specific MMPs), the activity differences have to be very large in order to be statistically significant. A full equation for the estimation of minimum significant activity difference, depending on the number of samples, standard deviation of the measurements and the true variance of the biochemical effect is developed. The influence of consistency of assay setups can directly be quantified via the variability and practical consequences for MMP analysis will be presented.
